# Multivariate regression analysis of outcomes following orthotopic liver transplantation in decompensated cirrhotics transplanted from the ICU

**DOI:** 10.1186/cc11001

**Published:** 2012-03-20

**Authors:** T Hughes, M McPhail, M Al-Freah, D Abeles, W Bernal, G Auzinger, J Wendon, C Willars

**Affiliations:** 1 Institute of Liver Studies, King's College Hospital, London, UK

## Introduction

Patients listed for orthotopic liver transplantation (OLT) frequently develop complications resulting in transfer to the ICUs of tertiary centres. The ICU mortality for cirrhotics has been variously reported from 38% to in excess of 90% [1]. The APACHE II score, MELD score and bacteraemia are independent predictors of mortality [2]. The aim of this study was to identify the risk factors relating to early mortality after OLT in cirrhotics transplanted from the ICU.

## Methods

A retrospective analysis of 1,284 patients transplanted between the dates of 1 January 2000 and 31 December 2008 in a major UK liver transplant centre was performed. Patient characteristics were recorded at transplant assessment and on the day of transplant: age, MELD score, UKELD score, serum sodium, creatinine, bilirubin, albumin, INR. Organ support (including ventilation, inotropic support and haemofiltration), lactate and APACHE II score on ICU admission and at the time of transplantation were also analysed. The primary outcome measure was patient survival at 3 months. Statistical analysis was by Mann-Whitney test, logistic regression and area under the receiver-operator curve analysis.

## Results

Eighty-one patients were transplanted from the ICU with cirrhosis complications. Statistical significance was demonstrated for admission lactate (*P *= 0.032), transplant lactate (*P *< 0.000), transplant APACHE II score (*P *= 0.001), admission inotropic support (*P *= 0.019), transplant inotropic support (*P *< 0.000) and transplant renal support (*P *< 0.000) when comparing 3-month survival with death on univariate analysis. On multivariate logistic regression analysis, high lactate (OR 1.28, 95% CI 1.08 to 1.51, *P *= 0.003) and use of renal replacement therapy (OR 3.52, 95% CI 1.42 to 8.74, *P *= 0.006) at the time of transplantation were independently associated with poor outcome. A combination of these two measures had an AUROC of 0.883 (0.791 to 0.945, *P *< 0.001, sensitivity 86%, specificity 86%) for prediction of death within 3 months.

## Conclusion

Patients with chronic liver disease transplanted from the ICU have a worse outcome if they require renal support or demonstrate hyperlactataemia on the day of transplantation.
